# Comparative Analysis of Soybean Root Proteome Reveals Molecular Basis of Differential Carboxylate Efflux under Low Phosphorus Stress

**DOI:** 10.3390/genes8120341

**Published:** 2017-11-30

**Authors:** Krishnapriya Vengavasi, Renu Pandey, Gerard Abraham, Ravindra Kumar Yadav

**Affiliations:** 1 Mineral Nutrition Laboratory, Division of Plant Physiology, ICAR-Indian Agricultural Research Institute, New Delhi 110012, India; krishnapriya19@gmail.com; 2 National Centre for Conservation and Utilization of Blue Green Algae, Division of Microbiology, ICAR-Indian Agricultural Research Institute, New Delhi 110012, India; gabraham@iari.res.in (G.A.); ravindrabga@gmail.com (R.K.Y.)

**Keywords:** carboxylate efflux, gene expression analysis, *Glycine max*, mass spectrometry, phosphorus stress, root proteome, two-dimensional gel electrophoresis

## Abstract

Carboxylate efflux from roots is a crucial and differential response of soybean genotypes to low phosphorus (P) stress. Exudation of carboxylic acids including oxalate, citrate, succinate and fumarate was induced under low P stress, particularly in P-efficient soybean genotypes. Enhancement of root length, surface area and volume further improved P acquisition under low P stress. To understand the molecular basis of carboxylate efflux under low P stress, the root proteome of contrasting genotypes (P-efficient: EC-232019 and P-inefficient: EC-113396) was compared. Among a total of 325 spots, 105 (32%) were differentially abundant proteins (DAPs) between sufficient (250 µM) and low P (4 µM) levels. Abundance of 44 (14%) proteins decreased by more than two-fold under low P stress, while 61 (19%) proteins increased by more than two-fold. Protein identification and annotation revealed that the DAPs were involved in a myriad of functions including carboxylic acid synthesis, carbohydrate, protein and lipid metabolism. Proteins with significant abundance included malate dehydrogenase, isocitrate dehydrogenase, phosphoglucomutase, phosphoglycerate mutase, fructokinase, enolase, phosphoglycerate kinase, triosephosphate isomerase, alcohol dehydrogenase, glucan water dikinase, glutamine synthetase and argininosuccinate lyase. Inferences from proteomic analysis suggests the crosstalk between various metabolic pathways implicated in conferring superior P acquisition efficiency under stress.

## 1. Introduction

Phosphorus (P) is an essential element for plant growth and development, with structural (nucleic acids, phospholipids), metabolic (energy transfer) and regulatory functions. P nutrition positively affects crop growth, with significant influence on above- (leaf area, dry matter accumulation, leaf P content, photosynthesis) and below-ground (root morphological traits, root exudation, symbiotic association) processes [1]. In soybean (*Glycine max* (L.) Merr.), P nutrition is important owing to its direct effect on growth and morphological responses and indirectly influencing nodulation and N_2_-fixation, and ultimately yield. Cultivating P-efficient soybean genotypes that can utilize the unavailable forms of soil P might be a sustainable option to increase crop productivity in the face of dwindling P reserves.

Altered root architecture and morphology, along with increased synthesis and excretion of carboxylic acids is crucial to enhance P acquisition [2,3,4]. Carboxylate ions release P from Al-P/Fe-P/Ca-P complexes by ligand exchange [5]. Low P stressed alfalfa (*Medicago sativa*) showed higher exudation of citrate, malate and fumarate ions compared to P sufficient plants [6]. Substantial amounts of malonic, succinic, fumaric, malic and citric acid were detected in root exudate of several P starved legumes [7]. Higher exudation of malate, oxalate and citrate exhibited by P-efficient soybean genotypes during P deficiency resulted in better growth traits compared to P-inefficient ones [8]. Higher citrate exudation helped P-efficient rice genotypes to mobilize P from sparingly soluble sources [9]. Root exudation in green gram (*Vigna radiata*) genotypes showed a positive association with biomass and P uptake [10]. 

Most common carboxylates in root exudates (citrate, malate, malonate, acetate, fumarate, succinate, lactate and oxalate) [11] are involved in respiration, stomatal regulation and other metabolic pathways, hence their efflux is primarily influenced by activity of glycolytic and tricarboxylic acid pathways [5]. Rate of exudation is a function of higher concentration of carboxylic acids in the cytosol as well as the expression and activation of efflux transporters/anion channels in the plasma membrane of root cells [12]. Members of the aluminum-activated malate transporter (ALMT) and multidrug and toxic compound extrusion (MATE) membrane protein families mediate malate and citrate efflux, respectively [13]. Earlier reports on efflux transporters in wheat (*Triticum aestivum*) TaALMT1 [14] and TaMATE1 [15] suggested that their expression was induced by aluminum toxicity. Overexpression of *TaALMT1* in barley improved P acquisition on acidic soils [16] confirming that P deficiency also induced expression of transporter proteins to facilitate carboxylate exudation. Liang and co-workers [17] isolated and characterized a malate efflux transporter from soybean *GmALMT1*, which was regulated by low pH, aluminum and P supply. In soybean, efflux transporters for malate (GmALMT1) and citrate (GmMATE) have been identified till date. Search for the missing players (genes/proteins) regulating synthesis and efflux of other carboxylic acids may enhance our knowledge in improving P uptake efficiency of soybean and related crops. 

Transcriptomic, proteomic and metabolomic analysis of different tissues subjected to varying levels and combinations of stress aid in identifying the molecular determinants of stress response mechanisms in plants. Among these, proteomic approaches are more robust as it is based on the expressed gene product. Proteome analyses to determine adaptation strategies to low P stress have been conducted in several crops such as maize (*Zea mays*) [18], oilseed rape (*Brassica napus*) [19] and *Arabidopsis thaliana* [20]. Comprehensive reference maps of soybean proteome [21,22,23] would accelerate the process of developing low P stress efficient soybean cultivars. Alterations to soybean proteome at the tissue and organelle level have been investigated under several abiotic stresses such as flooding, drought, salinity, aluminum and cadmium toxicity at different stages of crop growth [24,25]. However, influence of low P stress on soybean proteome, particularly in genotypes with contrasting root phenotypes and carboxylate exudation capacity has not been reported till date. To address this aspect, we conducted experiments employing a diverse panel of 116 soybean genotypes, to identify those with contrasting root exudation potential at low P compared to sufficient P [26]. Phenotyping for growth response and carboxylate efflux led to identification of EC-232019 (P-efficient) and EC-113396 (P-inefficient) with contrasting root exudation potential at low P stress [27]. In this paper, we present the results of comparative proteome analysis in root tissues of EC-232019 and EC-113396 grown at sufficient (250 μM) and low (4 μM) P level, which revealed several differentially abundant proteins (DAPs) under low P stress.

## 2. Materials and Methods

### 2.1. Plant Material and Growth Conditions

Soybean seeds of contrasting genotypes, EC-232019 (P-efficient) and EC-113396 (P-inefficient) were obtained from ICAR-Indian Institute of Soybean Research, Indore and Division of Genetics, ICAR-Indian Agricultural Research Institute, New Delhi, India, respectively. The seeds were surface sterilized with 0.1% (*w*/*v*) HgCl_2_ and wrapped in germination towels moistened with 1 mM CaCl_2_. Upon emergence of cotyledonary leaf, seedlings were transferred to nutrient solution with two P levels: sufficient (250 μM) and low P (4 μM). Seedlings were supported on a 5 cm thick styrofoam sheet at a spacing of 3 cm × 3 cm. The cotyledonary leaves were removed on third day of transfer to nutrient solution to minimize genotypic variation due to seed P content. Thirty-six such seedlings were accommodated in individual containers. Three replications with four seedlings each were maintained for all treatment combinations. Styrofoam sheet was placed in a plastic container with 10 L of basal nutrient solution. Composition of the nutrient solution was as described in Vengavasi et al. [27], and pH was maintained at 6.5 using either 1.0 N HCl or 1.0 N KOH. The solution was aerated continuously and renewed every third day. The experiment was conducted in greenhouse at the National Phytotron Facility, ICAR-IARI, New Delhi, India with day/night temperature of 30/26 °C, photoperiod of 12 h at a photon flux density of 850 μmol·m^−2^·s^−1^ and relative humidity of 85%. 

### 2.2. Growth Traits and Tissue Phosphorus Status

Roots of 20-d-old plants were scanned using root scanner (Regent Instruments Inc., Québec, QC, Canada) (Figure S1A) and the images were analyzed in WinRhizo Pro software (Regent Instruments, Ville de Québec, QC, Canada) to obtain total root length (cm plant^−1^), surface area (cm^2^ plant^−1^), volume (cm^3^ plant^−1^) and number of root tips. Root and shoot P concentration (μg g^−1^ dry weight (DW)) was determined by ascorbic acid method [28] after digestion of dried tissue with di-acid mixture (HNO_3_:HClO_3_::9:4). Tissue P content was calculated from P concentration in root or shoot, the sum of which was the total P uptake, expressed as μg P plant^−1^.

### 2.3. Collection and Quantification of Root Carboxylate Efflux

Twenty-d-old plants were placed in 100 mL Erlenmeyer flasks with their roots immersed well in 50 mL of trap solution (0.5 mM CaCl_2_, pH 4.5) (Figure S1B). The flasks were covered with black paper and exudates were collected from 08:00 to 12:00 h. Roots were washed in deionized water and blotted dry to determine fresh weight. The exudates were passed through Whatman no. 1 filter paper and frozen immediately to avoid microbial degradation. Root exudate was processed [26] and quantified through high-performance liquid chromatography (HPLC) (Agilent 1200 Infinity, Agilent Technologies, Palo Alto, CA, USA) with Hi-Plex H column as the stationary phase. The column temperature was set at 70 °C. Mobile phase (0.005 M H_2_SO_4_) was used at a flow rate of 0.6 mL min^−1^. Individual samples were run for 25 min and peaks captured by a refractive index detector with an optical temperature of 55 °C. Concentration of acids was quantified from the calibration curves of standards and expressed in μmol g^−1^ root fresh weight.

### 2.4. Protein Isolation and Quantification

Two g of root tissue was finely ground using liquid nitrogen in a pre-chilled pestle and mortar. Fifteen mL of extraction buffer containing 50 mM 2-[4-(2-hydroethyl)piperazin-1-yl] ethanesulfonic acid (HEPES) pH 7.5, 40% (*w*/*v*) sucrose and 0.1% (*v*/*v*) β-mercaptoethanol was added to the root powder and thoroughly homogenized. The homogenate was transferred to an Oak-ridge tube and 15 mL of phenol (equilibrated with 10 mM Tris pH 8.0 and 1 mM Ethylene diamine tetraacetic acid (EDTA) was added to it. After thorough mixing for 30 min, the homogenate was centrifuged at 5000 rpm for 10 min at 4 °C. Phenol phase was transferred to a fresh Oak-ridge tube to which 40 mL of precipitation buffer (0.1 M ammonium acetate in 100% (*v*/*v*) methanol) was added and incubated overnight at −20 °C. The protein pellet was obtained by centrifugation at 15,000 rpm for 30 min at 4 °C. The pellet was washed with 40 mL of 80% (*v*/*v*) acetone by vortexing and centrifuged at 15,000 rpm for 30 min at 4 °C. Washing step was repeated thrice after which the pellet was evaporated to dryness in a vacuum evaporator and stored at −80°C until further analysis.

For quantification of protein, the pellet was dissolved in a solubilization cocktail containing 7 M urea, 2 M thiourea, 2% (*w*/*v*) 3-[(3-Cholamidopropyl) dimethylammonio]-1-propanesulfonate hydrate (CHAPS), 400 mM dithiothreitol (DTT), and 1% (*v*/*v*) ampholyte (Biolyte pH 4–7). After 1 h of gentle stirring at room temperature, samples were centrifuged at 15,000 rpm for 30 min at 4 °C to remove the precipitated nucleic acids. Supernatants were transferred to fresh 1.5 mL Eppendorf tube and protein concentration was determined by Bradford method [29]. Protein quality was checked by subjecting all samples to sodium dodecyl sulfate polyacrylamide gel electrophoresis (SDS-PAGE) (Figure S2).

### 2.5. Two-Dimensional Gel Electrophoresis and Image Analysis

Immobilized pH gradient (IPG) strips (ReadyStrip^TM^, BioRad, Hercules, CA, USA) of 11.0 cm with pH 4 to 7 were passively rehydrated overnight with 400 µg of protein. Isoelectric focusing was performed in the Protean i12 IEF cell (BioRad, Hercules, CA, USA) with the following program: 200 volts for 180 min, 500 volts for 30 min, 1000 volts for 30 min, 6000 volts for 60 min, 6000 volts until a total of 3000 volt-hours followed by 500 volts for 20 h. After completion of isoelectric focusing, the strips were equilibrated for 15 min each in reducing buffer containing 50 mM Tris-HCl (pH 8.8), 6 M urea, 30% (*v*/*v*) glycerine, 2% (*w*/*v*) SDS and 20 mM DTT followed by alkylation buffer composed of 50 mM Tris-HCl (pH 8.8), 6 M urea, 30% (*v*/*v*) glycerine, 2% (*w*/*v*) SDS and 135 mM Iodoacetamide. Separating gel containing 12% (*w*/*v*) acrylamide, 375 mM Tris-HCl (pH 8.8), 0.1% (*w*/*v*) ammonium persulfate and 0.04% Tetramethylethylenediamine (TEMED) was poured into gel slabs. After polymerization, the equilibrated IPG strips were loaded on to the separating gel along with a protein ladder (11 to 245 kDa) placed at the corner well. The gels were overlaid with 1% (*w*/*v*) agarose (containing 0.1% (*w*/*v*) Bromophenol Blue tracking dye). Separation was performed at 13 °C with a constant current of 25 mA per gel in a Protean II xi cell (BioRad, Hercules, CA, USA) electrophoresis system. When tracking dye reached the base of the gel, the current was terminated. Gels were washed with deionized water and incubated overnight in staining solution containing 10% (*v*/*v*) acetic acid, 40% (*v*/*v*) methanol and 0.1% (*w*/*v*) Coomassie Brilliant Blue G-250. After staining, the gels were placed in destaining solution containing 7% (*v*/*v*) acetic acid and 25% (*v*/*v*) methanol until the background was clear and protein spots visible.

Image of gels were digitized in a densitometer (ImageScanner III, GE Healthcare Bio-Sciences, Uppsala, Sweden) for analysis based on spot density and location (Figure S3). Image analysis was performed with PDQuest software version 8.0.1 (BioRad, Hercules, CA, USA). Spots were quantified on the basis of their relative volume which was determined by the ratio of the volume of a single spot to the whole set of spots under low P stress. Spots with significant (more than two-fold differential expression, α = 0.05 by Student’s *t*-test) and reproducible changes in three replicates were used for further analysis.

### 2.6. Trypsin Digestion of Proteins

The protein spots of interest were picked from the gels, washed twice with MilliQ water, followed by 50% (*v*/*v*) acetonitrile. To this, 100 µL of 10 mM DTT dissolved in 25 mM ammonium bicarbonate (reducing buffer) was added and incubated at 45 °C for 60 min. Reducing buffer was discarded and protein spots were incubated in dark for 15 min with 100 µL of 10 mM iodoacetamide dissolved in 25 mM ammonium bicarbonate (alkylating buffer). This was followed by two sequential washing steps with 50% (*v*/*v*) and 100% (*v*/*v*) acetonitrile. The pellet was allowed to dry thoroughly and resuspended in 50 µL of 10 µg mL^−1^ trypsin dissolved in 25 mM ammonium bicarbonate and incubated overnight at 35 °C. The supernatant was transferred to a fresh Eppendorf tube and lyophilized at −80 °C. Lyophilized peptides were suspended in 1% (*w*/*v*) tetrafluoroacetic acid in 50% (*v*/*v*) acetonitrile prior to mass spectrometry.

### 2.7. Mass Spectrometry for Protein Identification

The peptide mass fingerprints of DAPs were collected on an AB Sciex TOF/TOF™ 5800 system with Series Explorer™ 7000 (AB Sciex, Concord, ON, Canada). The parameters were set as follows: Digestion enzyme: trypsin (specificity: C-terminal to Arginine and Lysine) with one missed cleavage; fixed modification: Carbamidomethyl (C); Mass Spectrometry (precursor-ion) peak filtering: 800–4000 m/z interval, monoisotopic, minimum signal-to-noise ratio (S/N) 10, mass tolerance 250 ppm; database used: Viridiplantae taxonomic sub-database (52,74,071 sequences) of the National Centre for Biotechnology Information protein database (NCBIprot, release date 08-11-2017; 12,86,24,863 sequences). Peptide mass fingerprints were searched against the database using the online Mascot server (version 2.6.0, Matrix Science Limited, London, UK). Proteins with Mascot score greater than 80 were considered as significant (*P* < 0.05) hits. 

### 2.8. In-Silico Analysis for Protein Annotation

Protein sequences identified from the Mascot search were functionally annotated using the bioinformatics platform Blast2GO [30] by assigning their associated generic Gene Ontology (GO) terms and Enzyme codes. This was based on homology to proteins from other species as determined by BLAST and the occurrence of InterPro functional domains identified by InterProScan. Annotations were further expanded using ANNEX [31]. BLAST searches were conducted for each protein (TBLASTX, nr database, report 20 hits, maximum e-value 1E^−10^), followed by mapping and annotation. The resulting GO terms were mapped onto the corresponding Plant GO Slim terms. The annotated enzymes were also assigned to their respective KEGG pathway maps [32].

### 2.9. Validation of DAPs at Transcript Level by Reverse Transcription-qPCR

Total RNA was isolated from root tissue with TRIzol reagent (Invitrogen) and quantified in Nanodrop1000 Spectrophotometer (Thermo Scientific, Waltham, MA, USA). Contamination by genomic DNA was removed by treating 10 μg of RNA with DNase I (Promega). RNA integrity was verified in 1% (*w*/*v*) agarose gel stained with ethidium bromide and visualized under UV light in a gel documentation system (AlphaImager, Cell Biosciences, Heidelberg, Germany). cDNA was synthesized using SuperScript III reverse transcriptase (Invitrogen) according to the manufacturer’s instructions. All samples were normalized to contain 50 ng·μL^−1^ of cDNA confirmed by the amplification of the reference gene “elongation factor 1α” (*Gm_EF1*α) and visualized on a 3% (*w*/*v*) agarose gel. Gene-specific primers (Table S1A,B) of proteins with significant Mascot scores were designed using the RealTime quantitative polymerase chain reaction (qPCR) tool of Integrated DNA Technologies [33]. Reverse transcription quantitative polymerase chain reaction (RT-qPCR) was carried out using the DyNAmoColorFlash SYBR Green I qPCR kit (Thermo Scientific, Waltham, MA, USA) on a Stratagene Mx3005P qPCR System (Agilent Technologies, Santa Clara, CA, USA). Reverse transcription-qPCR was performed in triplicate of 20 μL reaction containing 10 μL of 2X SYBR Green I master mix, 0.12 μL of 50X ROX passive reference dye, 1 μL of forward primer (0.5 pmol μL^−1^), 1 μL of reverse primer (0.5 pmol μL^−1^), 1 μL of cDNA template and 6.88 μL of nuclease-free water. The cycling parameters were as follows: 10 min of pre-denaturation at 95 °C, 40 cycles of 30 s at 95 °C and 30 s at 60 °C, followed by dissociation curve analysis (1 min at 95 °C, 30 s at 55 °C and ramp up to 95 °C). The MxPro software version 4.10 (Agilent Technologies, Santa Clara, CA, USA) was used for data collection. Melt curves were examined to detect inadvertent multiple amplicons. Primer specificity was also ensured by presence of single PCR product visualized on a 3% (*w*/*v*) agarose gel. The comparative cycle threshold method [34] was used to calculate relative transcript levels at experimental condition (low P) with three reference genes: 60S ribosomal subunit (*Gm_60S*), F-box (*Gm_F-box*) and *GmEF1*α. Negative controls were incorporated for each primer pair and individual PCR reactions were performed in triplicates.

### 2.10. Experimental Design and Statistical Rationale

All experiments were completely randomized with two factors: phosphorus level (P) and genotype (G). Physiological experiments to measure growth, root traits and root exudation were technically replicated thrice (*n* = 9), while protein isolation and 2D separation was carried out once in each biological replicate (*n* = 3). For validation of gene expression, RNA was isolated from three biological replicates, and RT-qPCR was carried out in three technical replicates (*n* = 9). Procedures for descriptive statistics and analysis of variance (α = 0.001 for physiological data; α = 0.05 for proteomics and expression validation) were carried out in the statistical software R version 3.1.2 (R Foundation for Statistical Computing, Vienna, Austria). Graphs and figures were plotted using GraphPad Prism version 6.00 (GraphPad Software, La Jolla, CA, USA).

## 3. Results

Soybean genotypes EC-232019 and EC-113396 responded differentially to low P stress, in terms of biomass accumulation, root system traits, uptake of P and carboxylate efflux as well as the root proteome profile. The salient results of the investigation are highlighted below.

### 3.1. Biomass Accumulation, Root System Traits and Tissue PosphorusStatus

**Figure 1:**
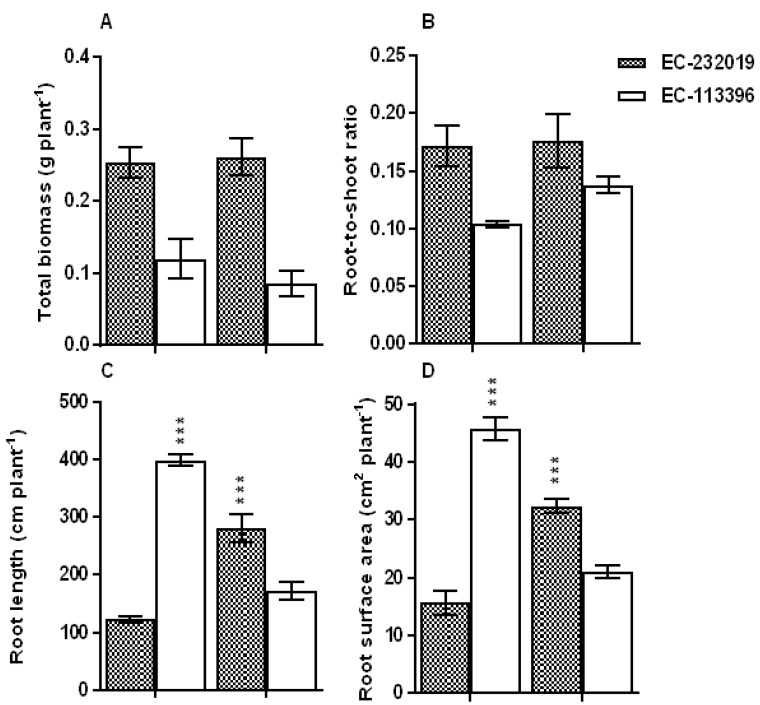

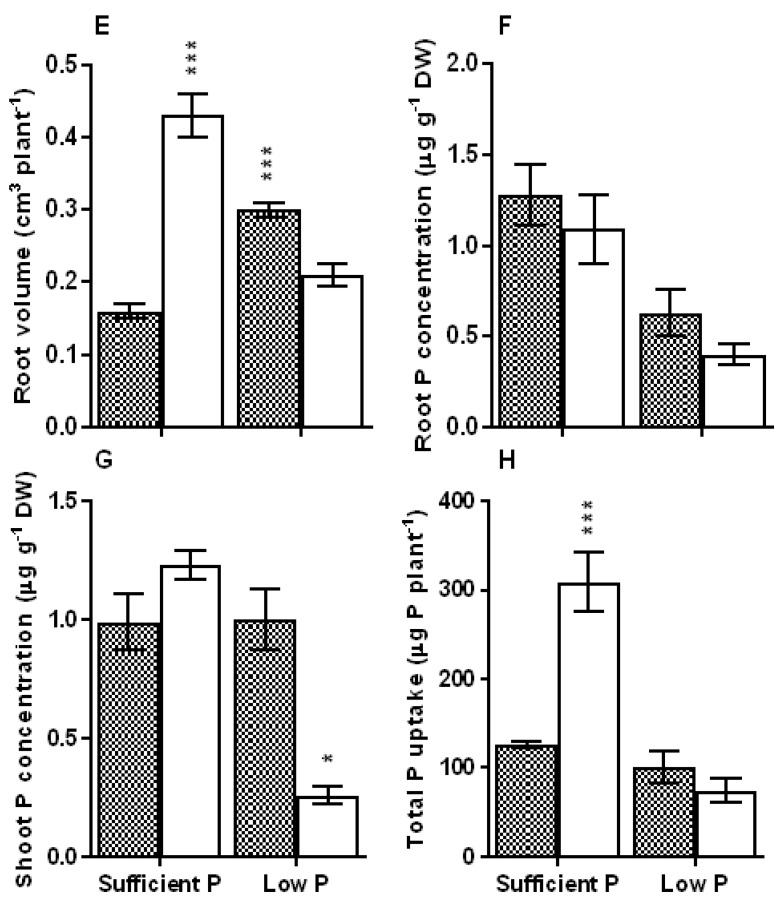
Variation in (**A**) total biomass, (**B**) root-to-shoot ratio, (**C**) root length, (**D**) root surface area, (**E**) root volume, (**F**) root phosphorus (P) concentration (**G**) shoot P concentration and (**H**) total P uptake in contrasting soybean genotypes grown at sufficient (250 μM) and low (4 μM) P. Data correspond to mean ± Standard Error (*n* = 9). DW: dry weight. *, ** and *** denote significance at 0.05, 0.01 and 0.001 probability levels, respectively.

P level, genotype and interactive effect of P × genotype on total biomass and root-to-shoot ratio was significant (*P* < 0.001) (Table S2). Reduction in biomass was more prominent in EC-113396 compared to EC-232019 (Figure 1A). Biomass partitioning to the roots was higher in EC-232019 compared to EC-113396 under both sufficient and low P level (Figure 1B). P level had no significant effect on root-to-shoot ratio EC-232019, while there was a slight increase under low P in EC-113396. Significant genotypic variation was observed in root length, surface area and volume under low P stress (Table S2, Figure 1C–E). Low P stress increased root surface area by 106% and root volume by 85% in the EC-232019 whereas both traits were reduced by 50% in EC-113396 at low P compared to sufficient P. P level significantly (P < 0.001) influenced tissue P status, with an overall reduction in shoot (63%) and root (73%) P concentrations at low P stress compared to sufficient P (Table S2, Figure 1F,G) in both genotypes. Irrespective of genotype, total P uptake was 70% lower at low P as compared to sufficient P (Figure 1H), while EC-232019 exhibited least reduction (19%) in total P uptake at low P compared to sufficient P.

### 3.2. Carboxylate Efflux in Response to Low Posphorus Stress

**Figure 2:**
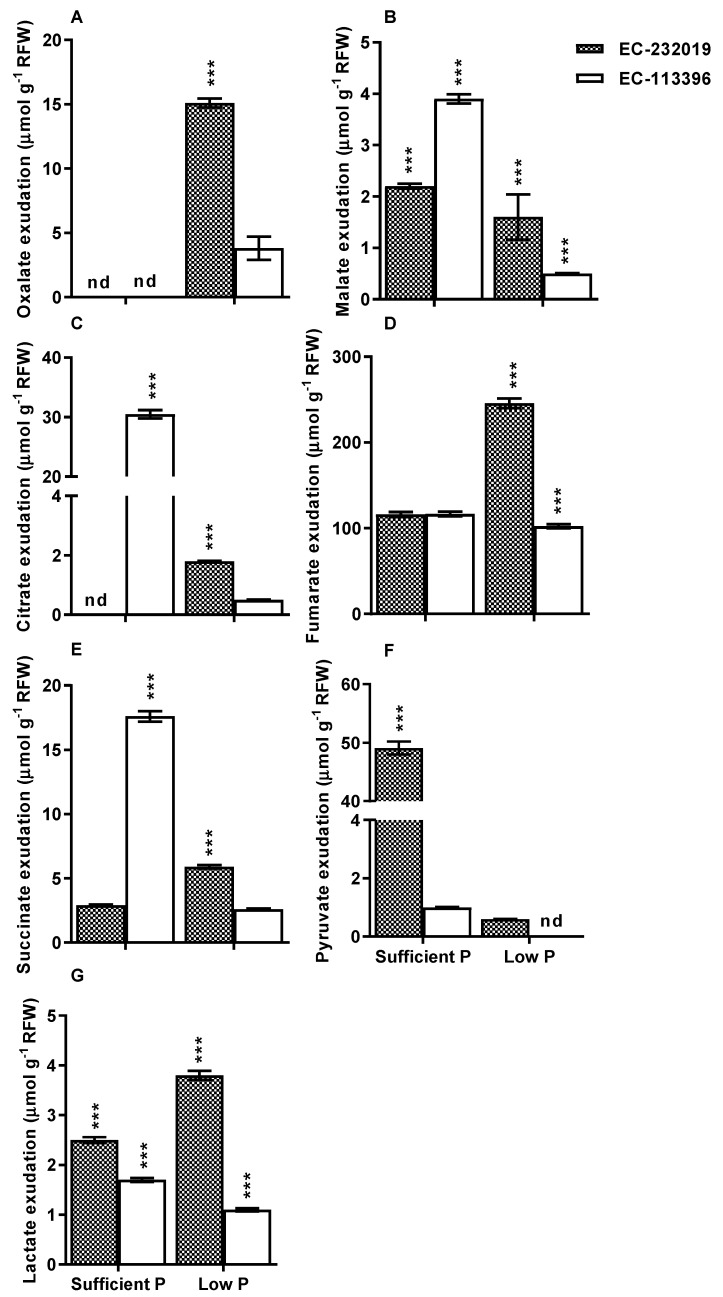
Variation in root exudation of (**A**) oxalate, (**B**) malate, (**C**) citrate, (**D**) fumarate, (**E**) succinate, (**F**) pyruvate and (**G**) lactate in contrasting soybean genotypes grown at sufficient (250 μM) and low (4 μM) P. Data correspond to mean±SE (*n* = 9). “nd” denotes peak “not detectable”. RFW: Root fresh weight. *, ** and *** denote significance at 0.05, 0.01 and 0.001 probability levels, respectively.

Low P stress-induced carboxylate efflux differed both in type and quantity, and exhibited a significant (*P* < 0.001) variability among contrasting soybean genotypes (Table S2). Averaged over P levels, EC-232019 exhibited higher rate of total carboxylate efflux compared to EC-113396. Low P induced-total carboxylate exudation increased by 58% in EC-232019, while it was reduced by 35% in EC-113396. In EC-232019, the efflux of oxalate, citrate, fumarate, succinate and lactate was induced under low P (Figure 2). Further, succinate and fumarate concentration in the root exudate increased by two-fold at low P in comparison to sufficient P in EC-232019.

### 3.3. Comparative Analysis of Soybean Root Proteome

**Figure 3:**
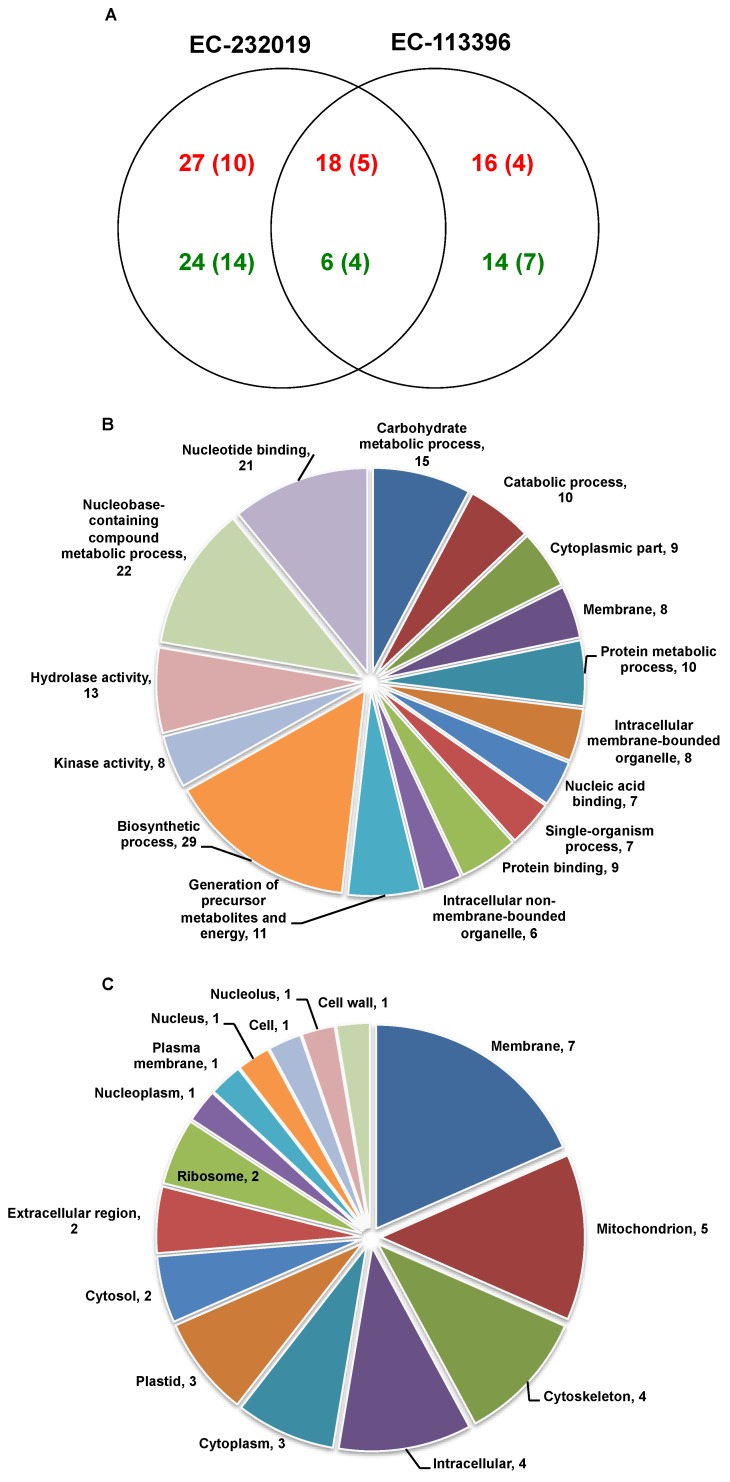
(**A**) Venn diagram showing number of differentially abundant proteins on two-dimensional electrophoretic gels at low P condition in comparison to sufficient P. Number indicated in green color are decreased proteins at low P, while red color are increased proteins at low P. Numbers within parenthesis correspond to proteins with significant Mascot score. Distribution of differentially abundant proteins based on gene ontology with respect to (**B**) biological process and (**C**) cellular localization.

Staining of two-dimensional electrophoretic gels using Coomassie Brilliant Blue dye revealed a total of 325 protein spots in the root tissue of EC-232019 and EC-113396 at low and sufficient P (Figure S3). Out of these, 105 (32%) DAPs were observed between sufficient and low P levels (Figure 3A). A total of 44 (14%) proteins decreased by more than two-fold under low P stress, while 61 (19%) proteins increased by more than two-fold at low P. These 105 DAP spots were picked from the gels, digested with trypsin and sequenced by matrix-assisted laser desorption/ionization (MALDI) to obtain the peptide mass fingerprints. Annotated spectra of the DAP spots are provided in Datasets S1 and S2.

#### 3.3.1. Differentially Abundant Proteins in Response to Low Posphorus Stress

**Figure 4:**
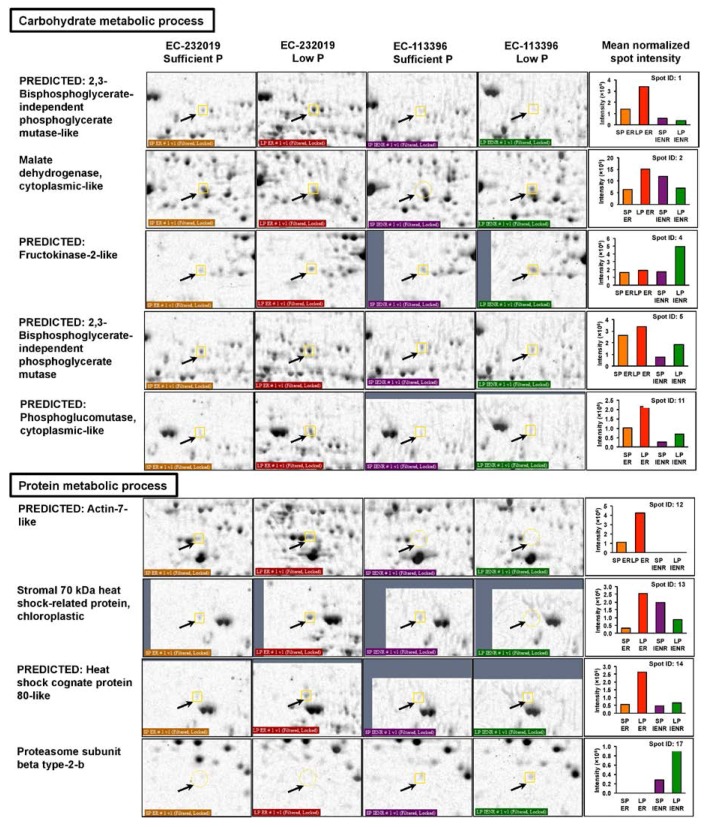

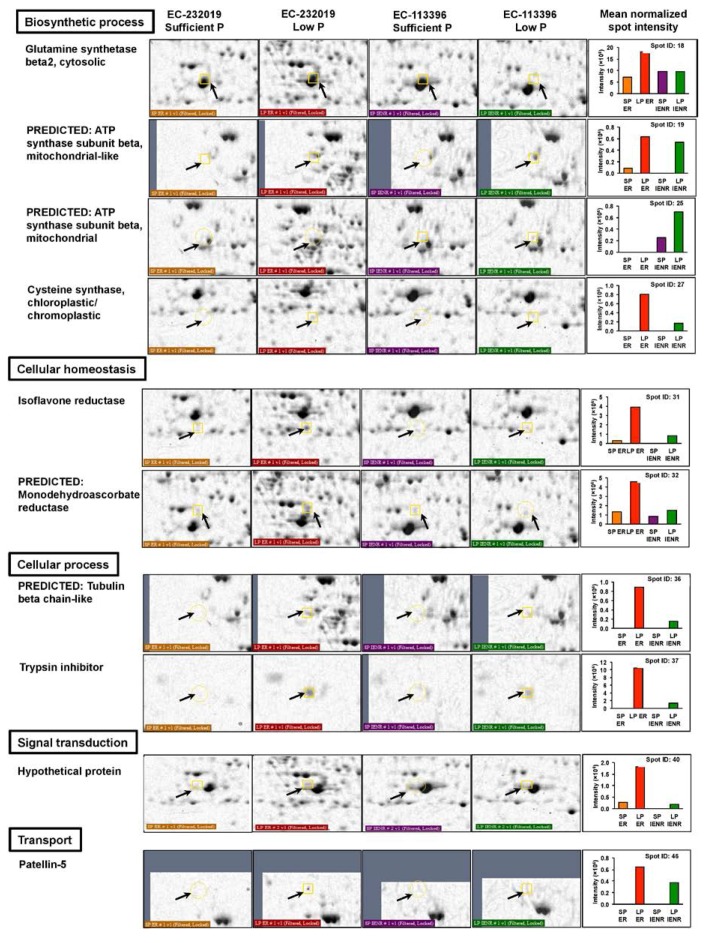
Root proteins increased by more than two-fold at low (4 μM) P in contrasting soybean genotypes. Arrows denote the protein spots zoomed for better visualization. Bars in the graph denote the mean (*n* = 3) normalized intensity of protein spots on gels of EC-232019 Sufficient P (orange, SP ER), EC-232019 Low P (red, LP ER), EC-113396 Sufficient P (violet, SP IENR) and EC-113396 Low P (green, LP IENR). Proteins with significant Mascot score are presented here. Refer Table 1 for entire list of increased proteins.

**Figure 5:**
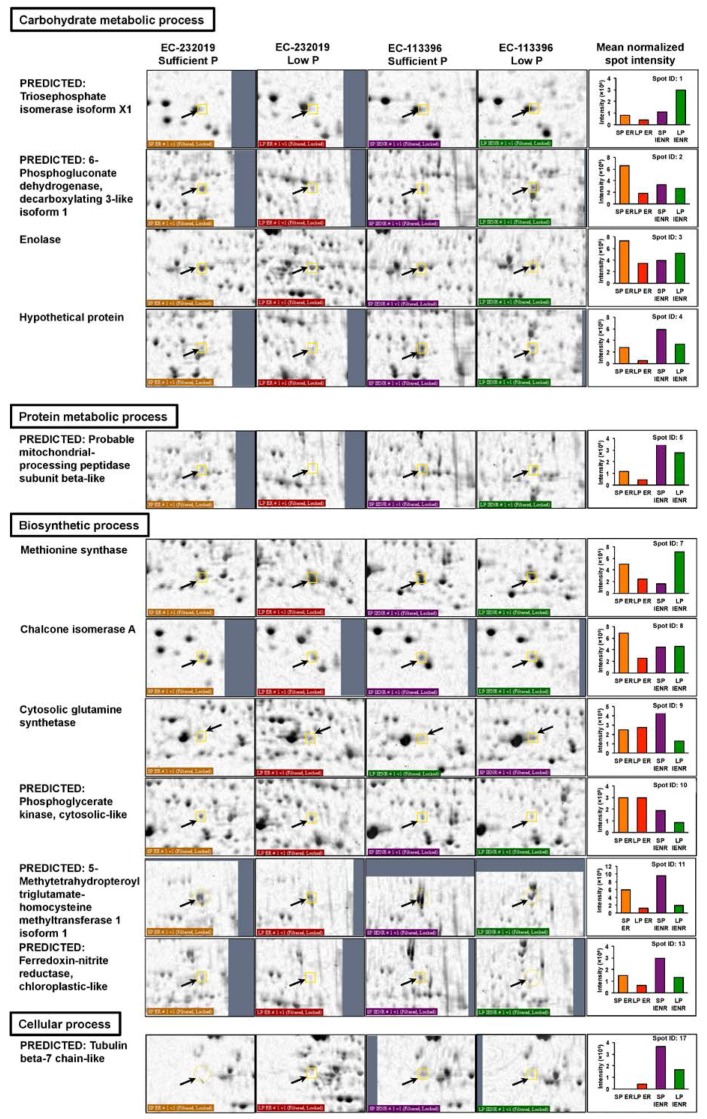

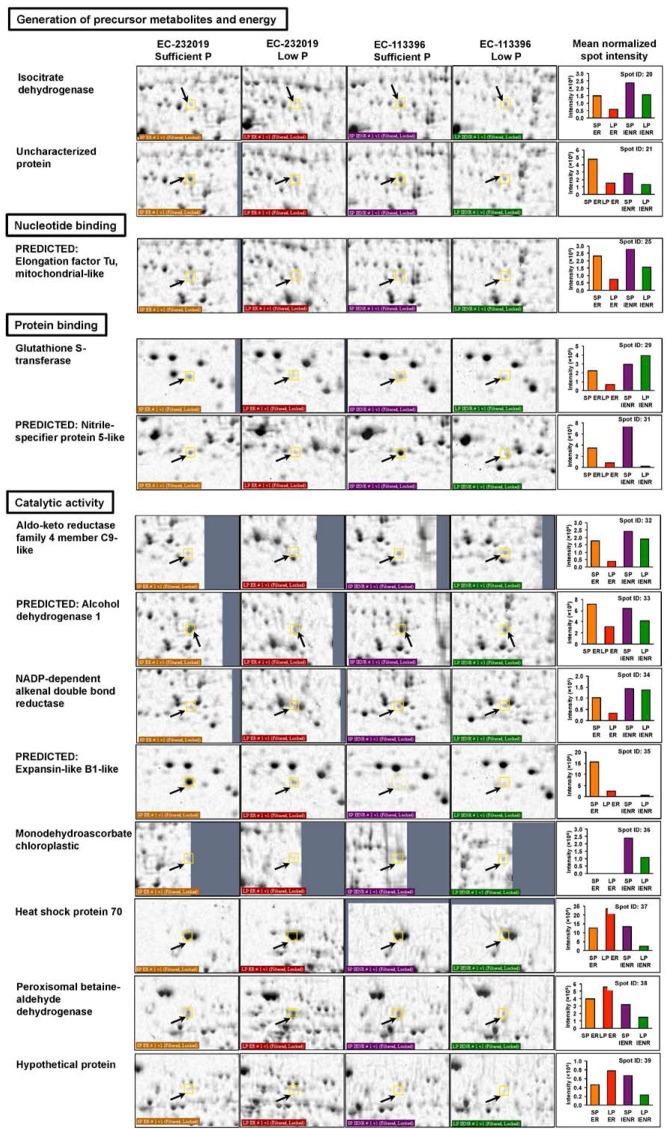
Root proteins decreased by more than two-fold at low (4 μM) P in contrasting soybean genotypes. Arrows denote the protein spots zoomed for better visualization. Bars in the graph denote the mean (*n* = 3) normalized intensity of protein spots on gels ofEC-232019 Sufficient P (orange, SP ER), EC-232019 Low P (red, LP ER), EC-113396 Sufficient P (violet, SP IENR) and EC-113396 Low P (green, LP IENR). Proteins with significant Mascot score are presented here. Refer Table 2 for entire list of decreased proteins.

Functional annotation of the DAPs based on gene ontology revealed their involvement in a myriad of biological processes including biosynthetic pathways, generation of precursor metabolites and energy, carbohydrate, protein and lipid metabolism (Figure 3B), localized to different cellular components (Figure 3C). Out of 61 increased proteins at low P, 27 were specific to EC-232019, 16 increased only in EC-113396 and 18 were common to both genotypes (Table 1). Eighteen peptide sequences with significant Mascot scores were derived from taxonomic databases of soybean (*G. max*) or its wild relative (*G. soja*). Some of the proteins significantly increased at low P condition were phosphoglycerate mutase, malate dehydrogenase, fructokinase, phosphoglucomutase, cysteine synthase, actin, 70 kDa heat shock-related protein, heat shock cognate protein, proteasome, ATP synthase, isoflavone reductase and monodehydroascorbate reductase (Figure 4). Among the proteins with decreased at low P, six were common to both genotypes, while 24 and 14 were specific to EC-232019 and EC-113396, respectively (Table 2). Twenty-three peptide sequences with significant Mascot scores were soybean (*G. max*) homologs. Twenty-two proteins were predicted from plant genome sequences, six were hypothetical and six uncharacterized or unknown proteins. Proteins decreased at low P included triosephosphate isomerase, phosphogluconate dehydrogenase, enolase, methionine synthase, chalcone isomerase, isocitrate dehydrogenase, glutathione-S-transferase, heat shock protein 70 kDa and alcohol dehydrogenase (Figure 5).

#### 3.3.2. Validation of Expression of Genes Encoding DAPs in Response to Low P Stress

**Figure 6:**
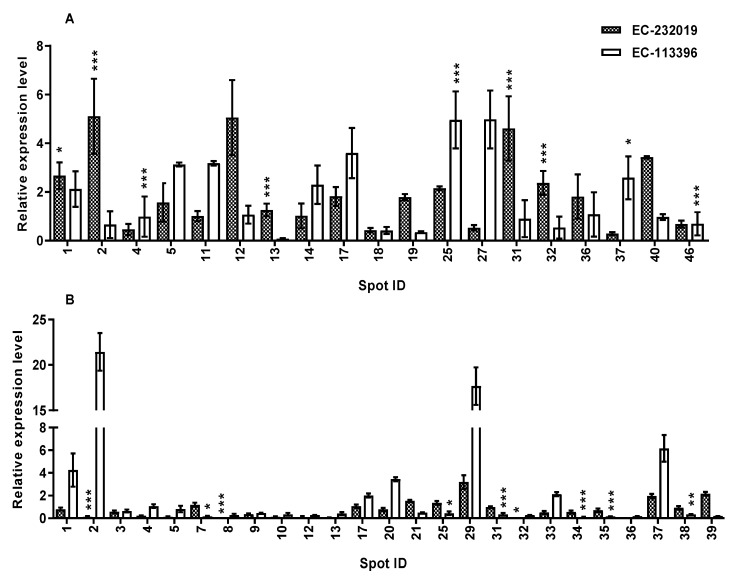
Relative transcript levels of genes encoding differentially abundant proteins (**A**) up-regulated and (**B**) down-regulated by more than two-fold at low (4 μM) P in soybean roots. Data correspond to mean±SE (*n* = 9). Spot ID corresponds to the protein spots in Supplemental Figure S3B,D. *, ** and *** denote significance at 0.05, 0.01 and 0.001 probability levels, respectively.

To validate the expression levels of genes encoding DAPs, RT-qPCR was performed on 44 out of the 105 proteins that showed differential expression under low P stress. In EC-232019, the genes encoding isoflavone reductase, actin-7-like, malate dehydrogenase and monodehydroascorbate reductase were up-regulated by more than two-fold at low P compared to sufficient P (Figure 6A, Table S3A). Transcripts of phosphoglucomutase, phosphoglycerate mutase, heat shock cognate protein 80-like, trypsin inhibitor, cysteine synthase and an uncharacterized protein showed more than two-fold expression at low P in EC-113396. The gene encoding ATP synthase β subunit was up-regulated by more than two-fold in both the genotypes. Contrary to protein expression, transcripts of the genes encoding fructokinase and cytosolic glutamine synthetase β_2_ were down-regulated at low P compared to sufficient P in both genotypes. Similarly, 19 genes were down-regulated at low P compared to sufficient P in agreement with protein expression pattern, whereas contrary to the proteome profile, transcripts of the genes encoding methionine synthase and glutathione S-transferase were up-regulated at low P compared to sufficient P in EC-232019 (Figure 6B, Table S3B). In summary, 36 genes displayed trends at the transcript level that were consistent with protein expression, while eight genes exhibited transcription trends that were opposite to that observed in the proteome profile.

## 4. Discussion

### 4.1. Soybean Genotypes Exhibit Improved Root System Traitsand Carboxylate Efflux under Low P Stress

Phosphorus nutrition had significant effect on the root system traits of both soybean genotypes, albeit the increase in root surface area and volume was augmented in the P-efficient EC-232019. Similar alterations to root morphology in response to low P stress was reported in lentil (*Lens culinaris*) [35], rapeseed [36], maize [37], green gram [10] and wheat [38]. P-efficient soybean genotype with improved root traits exhibited higher total P uptake under low P stress. The improved root system traits exhibited by the P-efficient soybean genotype EC-232019 attributed to the least reduction in total P uptake at low P compared to sufficient P. Higher root surface area improved P acquisition by increasing the amount of root exudates such as phosphatases, RNases, nucleases, apyrases and carboxylic acids [39]. Our results conform to responses of barley (*Hordeum vulgare*) to low P stress, wherein improved P uptake of efficient cultivars was attributed to enhanced root exudation that increased its ability to acquire more P [40]. P-efficient maize genotypes also exhibited higher carboxylic acid efflux, P uptake and biomass under low soil P availability in comparison to inefficient ones [41].

P-efficient soybean genotype EC-232019 exuded greater amounts of carboxylic acids under low P stress. Irrespective of P level or genotype, carboxylic acids in the root exudate comprised of fumarate >oxalate >lactate >pyruvate >succinate >malate. Other workers reported root exudates comprising fumarate, citrate and malate in P-stressed alfalfa [6] and soybean [7], which supported our findings. Oxalate and malate were the major carboxylic acids detected in P-efficient soybean [8,42]. Carboxylates mobilize P bound to metal ligands in the soil, hence their functionality depends on the number and arrangement of carboxyl and hydroxyl moieties. The complexing capacity for Al/Fe/Ca follows the decreasing order of tri- (citrate^3−^)> di- (malate^2−^, oxalate^2−^, fumarate^2−^, succinate^2^)> mono-carboxylic acids (lactate^1−^) [5]. Thus, soybean genotypes with higher root exudation potential efficiently maintain their tissue P status to sustain growth under low P stress condition.

### 4.2. Soybean Genotypes Exhibit Differential Molecular Regulation under Low Posphorus Stress

**Figure 7:**
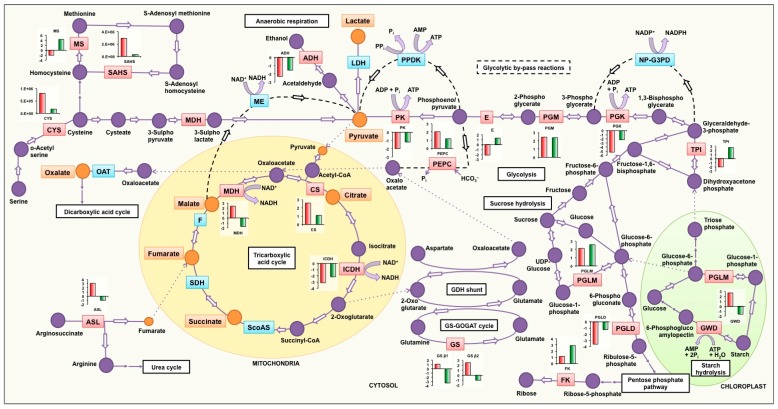
Model depicting involvement of several enzymes in carboxylic acid synthesis and glycolytic by-pass pathways functioning under low P stress in soybean roots. Carboxylic acids indicated in orange boxes were detected in the root exudates of soybean. Enzymes denoted in red boxes are differentially abundant under low P stress, relative fold change at low P compared to sufficient P in contrasting genotypes are denoted in red (EC-232019) and green (EC-113396) bars. Enzymes denoted in blue boxes might have a possible role in increasing carboxylic acid synthesis or glycolytic by-pass reactions under P stress. ADH: Alcohol dehydrogenase, ASL: Argininosuccinate lyase, CS: Citrate synthase, CYS: Cysteine synthase, E: Enolase, F: Fumarase, FK: Fructokinase, GDH: Glutamate dehydrogenase, GOGAT: Glutamate oxoglutarate amino transferase, GS: Glutamine synthetase, GWD: α-glucan water dikinase, ICDH: Isocitrate dehydrogenase, LDH: Lactate dehydrogenase, MDH: Malate dehydrogenase, ME: Malic enzyme, MS: Methionine synthase, NP-G3PD: Non-phosphorylating NADP dependent glyceraldehyde-3-phosphate dehydrogenase, OAT: Oxaloacetase, PEPC: Phospho*enol*pyruvate carboxylase, PGK: Phosphoglycerate kinase, PGLD: Phosphogluconate dehydrogenase, PGLM: Phosphoglucomutase, PGM: Phosphoglycerate mutase, PK: Pyruvate kinase, PPDK: Pyruvate phosphate dikinase, SAHS: S-adenosyl homocysteine synthase, ScoAS: Succinyl coenzyme A synthetase, SDH: Succinate dehydrogenase, TPI: Triosephosphate isomerase. PEPC, CS and PK data derived from enzyme activity (data published in Vengavasi et al. [26]).

Results of comparative proteome analysis and validation by RT-qPCR have been consolidated in Figure 7, which illustrates the differential molecular regulation in soybean genotypes with contrasting carboxylic acid synthesis and efflux under low P stress; suggesting the crosstalk between various metabolic pathways implicated in conferring superior P acquisition efficiency under stress.

#### 4.2.1. Tricarboxylic Acid Cycle and Glycolysis

Low P stress induced the expression of malate dehydrogenase in EC-232019 but not in EC-113396. Such a response at the molecular level might be attributed to increased synthesis and efflux of malate ions as observed in EC-232019 at low P. Further, abundance of isocitrate dehydrogenase decreased at low P (relative decline higher in EC-232019 compared to EC-113396), possibly leading to higher accumulation of malate in the root tissues [43]. 

Citrate synthase activity was higher at low P in EC-232019 (data published in Vengavasi et al. [26]), which might have increased the synthesis of citrate required for induction of efflux in the efficient genotype. Similarly, increase in the activity of phospho*enol*pyruvate carboxylase, one of the key regulatory enzymes for replenishment of carbon was higher in EC-232019 compared to EC-113396. Other enzymes in the glycolytic cycle including pyruvate kinase, enolase, phosphoglycerate kinase and triosephosphate isomerase decreased in EC-232019 under low P stress. Such a response in the P-efficient soybean genotype might indicate inorganic P recycling through alternative glycolytic by-pass reactions (dotted lines in Figure 7) catalyzed by nucleoside diphosphate kinase, non-phosphorylating NADP-glyceraldehyde-3-phosphate dehydrogenase, pyruvate phosphate dikinase, malic enzyme and phospho*enol*pyruvate carboxylase [44]. In concurrence, abundance of nucleoside diphosphate kinase increased at low P in EC-232019. 

#### 4.2.2. Starch Hydrolysis

Glucan water dikinase, an important enzyme involved in starch hydrolysis was increased by low P stress in EC-232019 but not in EC-113396, thereby increasing the flux of carbon through glycolysis and tricarboxylic acid cycle (TCA) cycle.

#### 4.2.3. Anaerobic Respiration

Alcohol dehydrogenase, the major enzyme producing ethanol from pyruvate decreased under low P stress in both genotypes, indicating the possible production of lactate ions, as evident from its higher efflux in soybean (Figure 2G).

#### 4.2.4. Other Anaplerotic Reactions Replenishing the TCA Cycle Intermediates

The increased expression of root cytosolic glutamine synthetase under low P in EC-232019 and not in EC-113396 suggests its role in stress tolerance, which is in agreement with results obtained in creeping bentgrass (*Agrostis stolonifera*) [45]. Reactions occurring in the glutamine synthetase-glutamine:2-oxoglutarate amidotransferase GS-GOGAT cycle and the glutamate dehydrogenase (GDH) shunt possibly siphoned carbon in amino acids back into the TCA cycle [46]. Argininosuccinate lyase that catalyzes the irreversible breakdown of argininosuccinate to arginine and fumarate increased under low P stress in EC-232019 and not in EC-113396. Fumarate synthesized in this reaction might be available for higher efflux at low P (evident from Figure 2D) in addition to that produced in the TCA cycle. Arginiosuccinate lyase is also involved in regulating root elongation as reported in rice [47]. Similarly, abundance of fructokinase, a major contributor to glycolytic carbon flux during root growth and development [48] also increased under low P in EC-113396.

#### 4.2.5. Synthesis of Sulfur Containing Amino Acids

In addition to regulation of carboxylic acid synthesis, low P stress in soybean influences several other metabolic pathways. Sustenance of sulfur assimilatory pathway under low P stress is important for recycling of P_i_ from plastidic phospholipids [49,50]. Increased abundance of the enzymes cysteine synthase, ATP sulfurylase and S-adenosyl homocysteine synthase is augmented in EC-232019, indicating sustained sulfur assimilation reactions even under low P stress. Methionine synthase, which is also involved in increasing the carbon source under stress as reported in creeping bentgrass [45], increased at low P in EC-113396 but not in EC-232019.

#### 4.2.6. Other Pathways Affected by Low P Stress

Abundance of proteins involved in maintenance of cellular homeostasis such as monodehydroascorbate reductase, 80 kDa heat shock cognate protein, 70 kDa heat shock related protein and proteasome subunit alpha and isoflavone reductase increased in EC-232019 under low P stress. Similar responses were observed in isopentenyl transferase (*ipt*) overexpressed creeping bentgrass tolerant to drought stress [45]. Isoflavone reductase-like gene (*OsIRL*) was found to be involved in scavenging reactive oxygen species in rice [51]. The abundance of actin increased at low P only in EC-232019, while tubulin increased in both genotypes under low P stress. Abundance of actin and tubulin proteins is reported under drought stress, with important roles in determining root cell structure [45]. Subunits of the energy producing protein ATP synthase showed variable response, being increased and decreased under low P in both genotypes.

### 4.3. Future Prospects

- Differentially abundant proteins with known physiological function may be tested rigorously for imparting P acquisition efficiency by creating overexpression/knockout mutant lines in soybean or other model systems.
- Hypothetical proteins with putative or unknown function identified in the proteomic analysis may be functionally characterized to ascertain their role(s) under low P stress.
- The identified genotypes may be potential donors in crop improvement programs to develop high-yielding P-efficient cultivars, an asset to low-input sustainable agriculture.

## 5. Conclusions

The proportion of carboxylates (including oxalate, citrate, succinate and fumarate) was highest among root exudates of P-efficient soybean EC-232019. Sustained growth of EC-232019 under low P stress may be attributed to improved root morphological traits and efflux of carboxylates. Key enzymes in the tricarboxylic acid cycle and glycolytic pathways that were induced or suppressed at low P stress showed clear-cut differential regulation among the contrasting genotypes. Enzymes including but not limited to malate dehydrogenase, isocitrate dehydrogenase, phospho*enol*pyruvate carboxylase, citrate synthase, glutamine synthetase, argininosuccinate lyase and alcohol dehydrogenase might be implicated in contributing to the additional carbon required for the carboxylate synthesis and efflux in EC-232019 under low P stress. Further, alterations at the transcript and protein level suggest the interdependence of various metabolic pathways conferring higher P acquisition efficiency to plants under stress.

## Figures and Tables

**Table 1:** Root proteins increased by more than two-fold at low (4 μM) phosphorus (P) in contrasting soybean genotypes.

Spot ID	Accession Number	Description	Taxonomic Database	E	M_r_ (kDa)	pI	Mascot Score	#P	C (%)
**Carbohydrate metabolic process**									
1^a^	XP_003534616.1	Predicted: 2,3-Bisphosphoglycerate-independent phosphoglycerate mutase-like	*Glycine max*	0.00013	61.1	5.51	106	10/10	31
2^a^	NP_001243291.1	Malate dehydrogenase, cytoplasmic-like	*Glycine max*	0.00017	35.5	5.91	105	8/12	38
3^a^	XP_018674024.1	Predicted: α-glucan water dikinase 2 isoform X2	*Musa acuminata subsp. malaccensis*	0.44	144.4	8.55	71	10/10	8
4^b^	XP_003537935.1	Predicted: Fructokinase-2-like	*Glycine max*	5.3 × 10^−12^	35.6	4.96	180	12/8	47
5^b^	XP_003552336.1	Predicted: 2,3-bisphosphoglycerate-independent phosphoglycerate mutase	*Glycine max*	2.6 × 10^−5^	61.1	5.58	113	10/10	25
6^b^	BAS92721.1	Predicted protein	*Oryza sativa Japonica*	0.17	14.1	10.27	75	5/15	34
7^b^	XP_001758882.1	Predicted: β-galactosyltransferase 7-like	*Physcomitrella patens*	0.44	45.1	8.25	71	6/9	16
8^b^	XP_003531483.1	Phosphoglycerate kinase isomerase, cytosolic	*Glycine max*	6.6	42.4	6.28	59	5/15	18
9^b^	XP_021745158.1	SW15-dependent HO expression protein 3-like	*Chenopodium quinoa*	3.0	21.2	10.00	62	6/14	28
10^b^	XP_019436544.1	Predicted: Fructokinase-2-like	*Lupinus angustifolius*	5.4	35.8	5.28	60	6/14	25
11^a,b^	XP_006580435.1	Predicted: Phosphoglucomutase, cytoplasmic-like	*Glycine max*	2.1 × 10^−5^	63.7	5.33	114	11/9	20
**Protein metabolic process**									
12^a^	XP_003525105.1	Predicted: Actin-7-like	*Glycine max*	5.3 × 10^−6^	41.9	5.37	120	10/10	38
13^a^	KHN39675.1	Stromal 70 kDa heat shock-related protein, chloroplastic	*Glycine soja*	0.0021	65.5	4.84	94	10/10	21
14^a^	XP_010063241.1	Predicted: Heat shock cognate protein 80-like	*Eucalyptus grandis*	0.016	80.8	4.94	85	9/11	11
15^a^	XP_006422243.1	Hypothetical: Proteasome subunit α type-2-a	*Citrus clementia*	0.12	21.6	7.88	76	6/14	31
16^a^	CDY43475.1	Predicted protein	*Brassica napus*	21.0	6.2	4.78	54	4/16	38
17^b^	XP_015954976.1	Proteasome subunit β type-2-b	*Arachis duranensis*	1.7 × 10^−5^	22.7	6.30	115	7/13	37
**Biosynthetic process**									
18^a^	NP_001242332.2	Glutamine synthetase β_2_, cytosolic	*Glycine max*	6.6 × 10^−6^	39.3	5.48	119	9/11	40
19^a^	XP_003555932.1	Predicted: ATP synthase subunit β, mitochondrial-like	*Glycine max*	0.00011	59.9	5.80	107	10/10	26
20^a^	XP_014514203.1	ATP synthase subunit, mitochondrial	*Vigna radiata var. radiata*	0.12	59.8	5.90	76	8/12	19
21^a^	XP_009122013.1	Argininosuccinate lyase	*Brassica rapa*	0.17	58.3	5.41	75	8/12	18
22^a^	AQK43314.1	Hypothetical: Nucleoside diphosphate kinase 1	*Glycine max*	0.11	44.0	9.56	77	8/12	20
23^a^	KHN48251.1	Adenosine kinase 2	*Glycine soja*	0.33	38.1	5.29	72	6/14	29
24^a^	XP_009394624.1	Uncharacterized protein	*Musa acuminata subsp. malaccensis*	1.1	64.9	9.87	67	8/12	16
25^b^	XP_003536650.1	Predicted: ATP synthase subunit β, mitochondrial	*Glycine max*	5.3 × 10^−11^	59.9	5.80	170	14/6	38
26^b^	KZM83344.1	Hypothetical protein	*Daucus carota subsp. sativus*	0.33	14.2	6.41	72	5/15	44
27^a,b^	KHN32353.1	Cysteine synthase, chloroplastic/chromoplastic	*Glycine soja*	0.014	37.3	5.67	86	7/13	27
28^a,b^	XP_020889182.1	Glutamine synthetase cytosolic isozyme 1-3-like	*Arabidopsis lyrata subsp. lyrata*	0.16	38.7	5.72	75	7/13	18
**Lipid metabolic process**									
29^a,b^	CDY19393.1	Hypothetical: Oxysterol-binding protein 1d	*Brassica napus*	0.34	88.7	6.07	72	9/11	11
30^a,b^	KXG23463.1	Hypothetical: 3-hydroxy-3-methylglutaryl-coenzyme a reductase 1-like	*Sorghum bicolor*	2e^+2^	9.8	5.88	44	3/9	24
**Cellular homeostasis**									
31^a^	NP_001236037.2	Isoflavone reductase	*Glycine max*	1.7 × 10^−11^	35.7	5.30	175	11/9	47
32^a^	XP_003557022.1	Predicted: Monodehydroascorbate reductase	*Glycine max*	0.057	47.1	5.49	80	7/13	21
**Nucleobase-containing compound metabolic process**									
33^a^	XP_010932834.1	Predicted: ATP sulfurylase 1, chloroplastic-like	*Elaeis guineensis*	23.0	53.8	9.36	54	6/9	8
**Cellular process**									
34^a^	KRH58847.1	Predicted: S-adenosyl-homocysteinase hydrolase	*Glycine max*	0.13	55.7	5.79	76	8/12	21
35^a^	KOM56630.1	Hypothetical: Mediator of RNA polymerase II transcription subunit 32	*Vigna angularis*	1.1	61.9	11.06	67	8/12	16
36^a,b^	XP_011039269.1	Predicted: Tubulin beta chain-like	*Populus euphratica*	0.0019	50.7	4.76	94	8/12	18
37^a,b^	NP_001237543.1	Trypsin inhibitor	*Glycine max*	0.0047	18.3	6.12	90	6/14	53
38^a,b^	OMO89133.1	Shoot gravitropism protein	*Corchorus capsularis*	0.34	105.8	5.32	72	10/10	9
**Generation of precursor metabolites and energy**									
39^a,b^	AGV54452.1	NADPH-specific isocitrate dehydrogenase	*Phaseolus vulgaris*	1.3	46.4	6.00	66	8/12	17
**Signal transduction**									
40^a^	OTG28653.1	Hypothetical protein	*Helianthus annuus*	0.028	91.3	5.20	83	9/11	12
41^a,b^	XP_002959041.1	Small ARF-related GTPase	*Volvox carteri f. nagariensis*	1.6e^+2^	20.1	6.74	45	4/16	30
**Nucleic acid/protein binding**									
42^b^	OWM88181.1	Hypothetical protein	*Punica granatum*	3.3	57.5	9.61	62	7/13	17
43^a,b^	GAU40109.1	Hypothetical protein	*Trifolium subterraneum*	0.46	53.1	6.19	71	7/13	14
44^a,b^	XP_019085342.1	Uncharacterized protein	*Camelina sativa*	0.48	101.2	9.42	70	7/13	7
**Translation**									
45^a,b^	AAY62839.1	Small ribosomal protein subunit 4, partial	*Haplohymenium triste*	0.092	22.3	10.17	78	6/14	33
**Transport**									
46^a,b^	KHN32468.1	Patellin-5	*Glycine soja*	0.024	35.2	8.73	84	7/13	18
**Transferase activity**									
47^b^	OIW11558.1	Hypothetical: Methyltransferase	*Lupinus angustifolius*	5.9	76.6	4.93	60	8/12	11
**Unknown**									
48^a^	XP_003064461.1	Predicted protein	*Micromonas pusilla*	0.11	46.7	9.84	77	8/12	19
49^a^	KDP45225.1	Hypothetical protein	*Jatropha curcas*	0.32	27.0	5.33	72	6/14	27
50^a^	XP_002953155.1	Hypothetical protein	*Volvox carteri f. nagariensis*	0.28	44.4	6.96	71	7/13	14
51^a^	OWM69200.1	Hypothetical protein	*Punica granatum*	3.1	76.6	7.72	62	6/11	8
52^a^	BAD15857.1	Hypothetical protein	*Oryza sativa Japonica*	5.2e^+2^	14.7	12.21	40	4/16	31
53^a^	XP_020159678.1	Predicted: Vacuolar-sorting receptor 1-like isoform X3	*Aegilopos tauschii subsp. Tauschii*	1.7e^+3^	25.5	5.57	35	3/14	8
54^b^	OAE31941.1	Hypothetical protein	*Marchantia polymorpha subsp. ruderalis*	0.34	18.0	7.90	72	6/14	25
55^b^	XP_017621778.1	Uncharacterized protein	*Gossypium arboreum*	0.31	18.1	11.28	72	6/14	39
56^b^	XP_018726619.1	Predicted: Disease resistance protein	*Eucalyptus grandis*	6.2	33.1	7.01	59	6/14	17
57^b^	XP_020253187.1	R3H and coiled-coil domain-containing protein	*Asparagus officinalis*	6.3	39.1	4.78	59	6/14	18
58^a,b^	XP_018450448.1	Lysine-specific demethylase	*Raphanus sativus*	7.4	99.2	6.12	58	6/14	7
59^a,b^	EPS63431.1	Hypothetical: Probable membrane-associated kinase regulator 1	*Genlisea aurea*	25.0	23.9	10.24	53	5/15	26
60^a,b^	KHG04359.1	Hypothetical protein	*Gossypium arboreum*	3.6	3.4	3.87	62	3/17	90
61^a,b^	XP_011087585.1	Uncharacterized protein	*Sesamum indicum*	2.1e^+2^	43.8	4.77	44	5/15	8

Spot ID corresponds to the protein spots in Supplemental Figure S3B,D. Superscripts ^a^ and ^b^ denote increased proteins at low P compared to sufficient P in EC-232019 and EC-113396, respectively. E denotes the expectation value, M_r_ and pI are monoisotopic mass and calculated isoelectric point, respectively. #P and C denote the number of matched/unmatched peptides and protein sequence coverage, respectively. Proteins with Mascot score > 80 are significant (*P* < 0.05).

**Table 2:** Root proteins decreased by more than two-fold at low (4 μM) P in contrasting soybean genotypes.

Spot ID	Accession Number	Description	Taxonomic Database	E	M_r_ (kDa)	pI	Mascot Score	#P	C(%)
**Carbohydrate metabolic process**									
1^a^	XP_003547334.1	Predicted: Triosephosphate isomerase isoform X1	*Glycine max*	5.3 × 10^−6^	27.4	5.87	120	9/11	36
2^a^	XP_003531895.1	Predicted: 6-Phosphogluconate dehydrogenase, decarboxylating 3-like isoform 1	*Glycine max*	5.3 × 10^−6^	53.8	6.11	120	11/9	31
3^a^	AAS18240.1	Enolase	*Glycine max*	0.017	48.0	5.31	85	9/11	23
4^a^	OQU84669.1	Hypothetical protein	*Sorghum bicolor*	0.028	4.0	10.04	83	4/16	94
**Protein metabolic process**									
5^a^	XP_003552094.1	Predicted: Probable mitochondrial-processing peptidase subunit β-like	*Glycine max*	1.1 × 10^−7^	58.8	6.49	137	12/8	27
6^a^	OMO96428.1	Hypothetical protein	*Glycine max*	0.24	85.7	5.22	73	9/11	11
**Biosynthetic process**									
7^a^	NP_001235794.1	Methionine synthase	*Glycine max*	3.3 × 10^−11^	84.4	5.93	172	15/5	25
8^a^	NP_001235219.1	Chalcone isomerase A	*Glycine max*	0.0046	23.3	6.23	91	7/13	41
9^b^	NP_001238531.2	Cytosolic glutamine synthetase	*Glycine max*	4.2 × 10^−5^	39.1	5.48	111	9/11	31
10^b^	XP_003546821.1	Predicted: Phosphoglycerate kinase, cytosolic-like	*Glycine max*	0.00074	42.4	5.48	98	9/11	28
11^b^	XP_016685735.1	L10-interacting MYB domain-containing protein-like, isoform X1	*Gossypium hirsutum*	0.14	36.0	5.96	76	7/13	20
12^a,b^	XP_003554033.1	Predicted: 5-Methytetrahydropteroyltriglutamate-homocysteine methyltransferase 1 isoform 1	*Glycine max*	4.2 × 10^−6^	84.4	8.73	121	12/8	23
13^a,b^	XP_003529397.1	Predicted: Ferredoxin-nitrite reductase, chloroplastic-like	*Glycine max*	0.036	66.5	5.97	82	9/11	14
14^a,b^	XP_016752752.1	Uncharacterized: ATP synthase CF_0_ subunit I	*Gossypium hirsutum*	0.29	26.4	6.31	73	7/13	21
**Nucleobase-containing compound metabolic process**									
15^a^	Gm_SSP8106^a^	Predicted protein	*Ostreococcus lucimarinus*	0.28	46.3	6.30	73	6/9	19
16^a,b^	XP_021735866.1	Uncharacterized: ATP sulfurylase 2	*Chenopodium quinoa*	2.6	92.0	5.49	63	7/13	8
**Cellular process**									
17^b^	XP_009141007.1	Predicted: Tubulin β-7 chain-like	*Brassica napus*	0.00017	51.2	4.73	105	9/11	22
18^b^	XP_016580376.1	Sulfite oxidase	*Capsicum annuum*	3.0	69.1	9.45	62	6/14	11
19^b^	OQU86022.1	Hypothetical: Kinesin heavy chain	*Sorghum bicolor*	3.5	14.6	9.35	62	5/15	48
**Generation of precursor metabolites and energy**									
20^a^	AAA33978.1	Isocitrate dehydrogenase	*Glycine max*	5.3 × 10^−9^	49.5	6.13	150	13/7	32
21^a,b^	NP_001241237.1	Uncharacterized protein	*Glycine max*	6.6 × 10^−9^	46.4	5.87	149	13/7	32
**Metabolic process**									
22^a^	XP_013467839.1	Pyridoxal-5′-phosphate-dependent enzyme family protein	*Medicago truncatula*	0.21	22.2	7.66	74	6/14	30
**Signal transduction**									
23^a^	XP_006584765.1	Predicted: Calcineurin B-like protein 2-like	*Glycine max*	4.0	23.9	4.84	61	5/15	22
**Nucleotide/Nucleic acid binding**									
24^a^	XP_009797404.1	Predicted: Glycine-rich RNA-binding protein 4	*Nicotiana sylvestris*	1.3	18.4	9.32	66	5/15	31
25^a^	NP_001276267.2	Predicted: Elongation factor Tu, mitochondrial-like	*Glycine max*	0.05	49.3	6.40	80	8/12	23
26^b^	XP_016674018.1	Predicted: Coiled-coil domain-containing protein 22 homolog isoform X2	*Gossypium hirsutum*	2e^+2^	56.1	4.93	44	5/8	8
**Transport**									
27^a^	XP_007509415.1	Unknown protein	*Bathycoccus prasinos*	0.86	84.2	5.04	68	7/13	10
28^a^	XP_021753870.1	Uncharacterized: ATP synthase CF_1_ α	*Chenopodium quinoa*	0.18	20.2	8.91	75	5/7	19
**Protein binding**									
29^a^	NP_001239642.1	Glutathione S-transferase	*Glycine max*	3.3 × 10^−5^	24.9	5.73	112	7/13	36
30^a^	XP_021316845.1	Acyl-binding domain-containing protein 5-like	*Sorghum bicolor*	0.19	77.1	8.85	74	9/11	19
31^a,b^	XP_003531506.1	Predicted: Nitrile-specifier protein 5-like	*Glycine max*	3.3 × 10^−15^	36.0	5.59	212	14/6	55
**Catalytic activity**									
32^a^	KHN43834.1	Aldo-keto reductase family 4 member C9-like	*Glycine max*	1.3 × 10^−9^	35.0	6.40	156	12/8	47
33^a^	NP_001340170.1	Predicted: Alcohol dehydrogenase 1	*Glycine max*	2.6 × 10^−5^	41.6	5.97	113	9/11	32
34^a^	KHN30856.1	NADP-dependent alkenal double bond reductase	*Glycine max*	0.036	38.0	5.94	82	7/13	25
35^a^	XP_003549946.1	Predicted: Expansin-like B1-like	*Glycine max*	0.00021	28.2	6.30	104	7/13	43
36^b^	KHN16790.1	Monodehydroascorbate chloroplastic	*Glycine max*	2.1 × 10^−5^	52.4	8.36	144	12/8	33
37^b^	XP_003521330.1	Heat shock protein 70	*Glycine max*	6.6 × 10^−7^	71.8	5.05	129	12/8	26
38^b^	NP_001234990.1	Peroxisomal betaine-aldehyde dehydrogenase	*Glycine max*	0.00021	55.4	5.23	104	10/10	17
39^b^	KCW74152.1	Hypothetical protein	*Eucalyptus grandis*	0.046	52.8	9.73	81	10/10	18
**Kinase activity**									
40^b^	ONH93257.1	Hypothetical: Serine threonine-protein kinase 19	*Prunus persica*	0.84	25.3	9.11	68	7/13	25
**Transferase activity**									
41^b^	XP_004968119.1	Predicted: o-linked n-acetylglucosamine transferase-like	*Setaria italica*	0.11	50.3	10.45	77	8/12	16
**Unknown**									
42^a^	XP_018476189.1	Predicted: RRP12-like protein	*Raphanus sativus*	0.44	141.5	8.88	71	9/11	11
43^a^	XP_016506991.1	Uncharacterized protein	*Nicotiana tabacum*	0.45	17.4	8.95	71	6/14	25
44^b^	XP_011040448.1	Uncharacterized protein	*Populus euphratica*	2.1	83.8	6.22	64	7/7	6

Spot ID corresponds to the protein spots in Supplemental Figure S3A,C. Superscripts ^a^ and ^b^ denote decreased proteins at low P compared to sufficient P in EC-232019 and EC-113396, respectively. E denotes the expectation value, M_r_ and pI are monoisotopic mass and calculated isoelectric point, respectively. #P and C denote the number of matched/unmatched peptides and protein sequence coverage, respectively. Proteins with Mascot score > 80 are significant (*P* < 0.05).

## Supplementary Materials

The following are available online at www.mdpi.com/2073-4425/8/12/341/s1. Figure S1: (A) Digitized root images in two contrasting soybean genotypes grown at sufficient (250 μM) and low (4 μM) P, (B) soybean plants immersed in trap solution for root exudate collection, Figure S2: SDS-PAGE gels of total root protein in contrasting soybean genotypes grown at sufficient (250 μM) and low (4 μM) P, Figure S3: Two-dimensional gels of total root protein in contrasting soybean genotypes grown at sufficient (250 μM) and low (4 μM) P, Table S1A: Primers used for RT-qPCR to validate transcript levels of increased proteins at low P (4 μM), Table S1B:Primers used for RT-qPCR to validate transcript levels of decreased proteins at low P (4 μM), Table S2: Significance of F values derived from analysis of variance for various physiological traits in contrasting soybean genotypes, Table S3A: Significance of F values derived from analysis of variance of transcript levels of proteins increased at low P (4 μM), Table S3B: Significance of F values derived from analysis of variance of transcript levels of proteins decreased at low P (4 μM), Dataset S1: Annotated spectra of increased proteins at low P (4 μM) in soybean, Dataset S2: Annotated spectra of decreased proteins at low P (4 μM) in soybean.
